# A patient decision aid for risk‐reducing surgery in premenopausal *BRCA1/2* mutation carriers: Development process and pilot testing

**DOI:** 10.1111/hex.12661

**Published:** 2017-12-27

**Authors:** Marline G. Harmsen, Miranda P. Steenbeek, Nicoline Hoogerbrugge, Helena C. van Doorn, Katja N. Gaarenstroom, M. Caroline Vos, Leon F.A.G. Massuger, Joanne A. de Hullu, Rosella P.M.G. Hermens

**Affiliations:** ^1^ Department of Obstetrics and Gynaecology Radboud University Medical Center Nijmegen The Netherlands; ^2^ Department of Human Genetics Radboud University Medical Center Nijmegen The Netherlands; ^3^ Department of Gynaecology Erasmus MC Cancer Clinic Rotterdam The Netherlands; ^4^ Department of Obstetrics and Gynaecology Leiden University Medical Centre Leiden The Netherlands; ^5^ Department of Obstetrics and Gynaecology Elisabeth‐Tweesteden Hospital Tilburg The Netherlands; ^6^ Scientific Institute for Quality of Healthcare Radboud University Medical Center Nijmegen The Netherlands

**Keywords:** ovarian cancer, patient decision aid, premature menopause, salpingectomy, salpingo‐oophorectomy

## Abstract

**Background:**

*BRCA1/2* mutation carriers’ choice between risk‐reducing salpingo‐oophorectomy (RRSO) and salpingectomy with delayed oophorectomy is very complex. Aim was to develop a patient decision aid that combines evidence with patient preferences to facilitate decision making.

**Design:**

Systematic development of a patient decision aid in an iterative process of prototype development, alpha testing by patients and clinicians and revisions using International Patient Decision Aid Standards (IPDAS) quality criteria. Information was based on the available literature and current guidelines. A multidisciplinary steering group supervised the process.

**Setting and participants:**

Pre‐menopausal *BRCA1/2* mutation carriers choosing between RRSO and salpingectomy with delayed oophorectomy in Family Cancer Clinics in the Netherlands.

**Main outcome measures:**

IPDAS quality criteria, relevance, usability, clarity.

**Results:**

The patient decision aid underwent four rounds of alpha testing and revisions. Finally, two paper decision aids were developed: one for *BRCA1* and one for *BRCA2*. They both contained a general introduction, three chapters and a step‐by‐step plan containing a personal value clarification worksheet. During alpha testing, risk communication and information about premature menopause and hormone therapy were the most revised items. The patient decision aids fulfil 37 of 43 (86%) IPDAS criteria for content and development process.

**Discussion and conclusions:**

Both *BRCA1/2* mutation carriers and professionals are willing to use or offer the developed patient decision aids for risk‐reducing surgery. The patient decision aids have been found clear, balanced and comprehensible. Future testing among patients facing the decision should point out its effectiveness in improving decision making.

## 1 INTRODUCTION

Women harbouring a germline mutation in the *BRCA1* and/or *BRCA2* genes have an increased risk of breast and ovarian cancer.^1^ The most effective way to diminish this ovarian cancer risk is removing fallopian tubes and ovaries during risk‐reducing salpingo‐oophorectomy (RRSO), preferably around the age of 40 years.^2^, ^3^ However, salpingo‐oophorectomy at that age results in acute onset of premature menopause with several short‐ and long‐term health consequences.^4^, ^5^, ^6^

Therefore, salpingectomy upon completion of childbearing with delayed oophorectomy has been proposed as alternative strategy to reduce ovarian cancer risk.^7^, ^8^, ^9^ This strategy is based on the growing evidence that (serous) ovarian cancer mainly originates from the fallopian tube,^10^, ^11^, ^12^ and is currently being investigated in a Dutch preference trial (NCT02321228).^13^ However, safety in terms of ovarian cancer risk has not been proven yet, which is the main disadvantage of this alternative strategy.

Because of different pros and cons of both strategies, the choice of trial participants between RRSO and salpingectomy with delayed oophorectomy is complex and highly personal. It is important to educate and empower these women in the decision‐making process. Previously, patient decision aids have been shown to increase knowledge, improve risk perception, lower decisional conflict, reduce proportions of people remaining undecided and can improve patient satisfaction.^14^ Interestingly, they also increase the number of patients who prefer conservative treatment options rather than invasive surgery.^14^

Currently, no patient decision aid that includes the relatively new option of salpingectomy with delayed oophorectomy is available to *BRCA1/2* mutation carriers, nor in Dutch, nor in English. In the literature, two randomized controlled trials evaluating patient decision aids for women at increased risk of ovarian cancer only included RRSO and ovarian screening as options and were developed more than 10 years ago.^15^, ^16^ Patient decision aids about RRSO in *BRCA1/2* mutation carriers do not exist in Dutch. To use English decision aids including the RRSO option, as OvDex, which have become available online during the last 5 years,^17^, ^18^ they would need to be extensively adjusted because of the additional option of salpingectomy with delayed oophorectomy. Moreover, although OvDex^18^ can be personalized depending on age, type of mutation and breast cancer history, this decision aid is already very long with only two options (surgery or no surgery) and only fulfils 4 of 9 International Patient Decision Aid Standards (IPDAS)^19^ criteria to lower the risk of making a biased decision. Another decision aid developed by Healthwise^17^ is short and convenient. However, the target population is not limited to *BRCA1/2* mutation carriers, and information about cancer risks is limited and not visually presented. Furthermore, these existing decision aids would need to be translated from English into Dutch to fit our population.

Therefore, the purpose of this study was to combine evidence with patient preferences in a tool that provides decision support for *BRCA1/2* mutation carriers who participate in the aforementioned preference trial that compares RRSO with salpingectomy and delayed oophorectomy. As a result, the target audience consists of all trial participants: pre‐menopausal *BRCA1/2* mutation carriers who completed childbearing, who are 25‐40 (*BRCA1*) or 25‐45 (*BRCA2*) years old, and who are currently not being treated for any malignancy. Three options are discussed in the decision aid. The first option is no risk‐reducing surgery. Although *BRCA1/2* mutation carriers are rarely reluctant to any kind of risk‐reducing surgery,^20^ this option was mentioned in our PtDA to complete the overview of options and to place the effects of the other two options in perspective. The second option is RRSO between 35‐40 years old (*BRCA1*) and 40‐45 years old (*BRCA2*), as currently recommended in national and international guidelines.^21^, ^22^ The third option is risk‐reducing salpingectomy upon completion of childbearing with delayed oophorectomy between 40‐45 (*BRCA1*) and 45‐50 years old (*BRCA2*), which has not been proven effective yet in terms of improved quality of life or safe in terms of ovarian cancer risk. To stick closely to the objective of the preference trial and to limit the length of the decision aid, the project group decided to focus on ovarian cancer risk management. Hence, information on breast surveillance and risk‐reducing mastectomy was not included.

## 2 MATERIALS AND METHODS

### 2.1 Development

**Figure 1:**
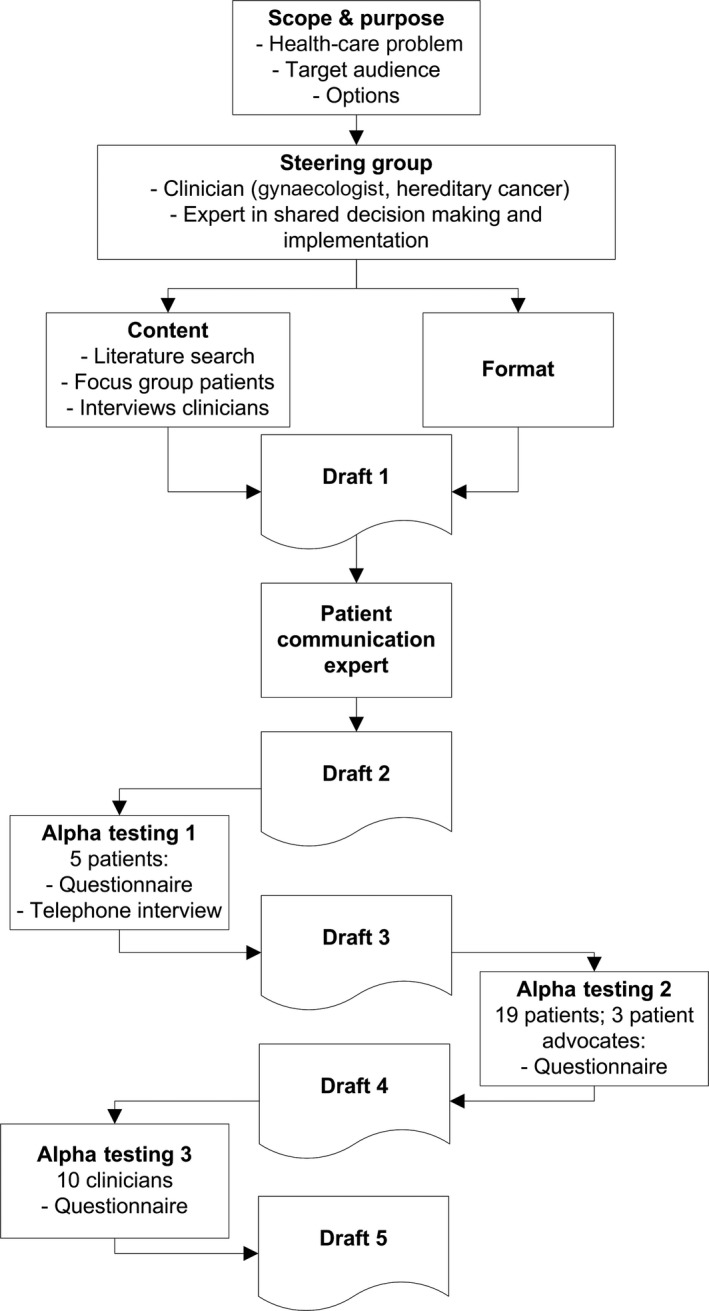
Systematic development process of the patient decision aid

Recommendations published by Coulter et al^23^ guided the development process of the patient decision aid (Figure 1). The prototype was developed by a project group consisting of a medical doctor, a gynaecologic oncologist and an expert in shared decision making and guideline implementation, assisted by a patient and professional expert panel, and in accordance with the international IPDAS criteria.^19^ The development process was supervised by a steering group, consisting of a professor of gynaecologic oncology, professor of hereditary cancer and biostatistician. None of the steering group members had any conflicts of interests.

#### 2.1.1 Scope and purpose

Given the complex decision that has to be made within our preference trial on RRSO and salpingectomy with delayed oophorectomy in *BRCA1/2* mutation carriers (NCT02321228),^13^ the project and steering group agreed on the need for a decision support tool for trial participants. Options to be discussed in the decision aid resulted from the options within the preference trial.^20^

#### 2.1.2 Exploring patients’ decisional needs

Points of consideration in the choice between RRSO and salpingectomy with delayed oophorectomy were extracted from the literature, searching the Medline database for “*BRCA*” or equivalents and “salpingectomy” or “delayed oophorectomy.” Furthermore, we interviewed 39 *BRCA1/2* mutation carriers and 23 of their health‐care professionals in the process of designing the aforementioned preference trial.^8^ The purpose of these interviews was to explore barriers and facilitators for the introduction of salpingectomy with delayed oophorectomy as alternative risk‐reducing strategy. One year later, a random selection of seven of these women attended another session to discuss the content of the trial patient information booklet and decision aid.

#### 2.1.3 Content and format

Based on the identified decisional needs, guidelines and expert opinions, the project group together with the steering group determined five domains that are important for decision making in this setting: (1) risks and (2) benefits of no surgery, salpingo‐oophorectomy and salpingectomy with delayed oophorectomy; (3) premature menopause and hormone replacement therapy (HRT); (4) strength and availability of evidence; and (5) women's preferences. For the first three domains, we collected available literature to compose the content of the decision aid; the fourth domain was based on appraisal of the evidence found. The literature search for domains (1) and (2) is described in detail elsewhere.^24^ The Medline database was searched for the third domain using the terms “*BRCA*” or equivalents and “menopause” or equivalents in September 2014. For the fifth domain, the best way to clarify individual preferences was also extracted from the literature and combined with expert opinions within our project group. To be able to hand out and discuss the patient decision aid at the outpatient department, the patient decision aid was developed as print booklet that can be used complimentary to face‐to‐face counselling. The booklet is also a good format to be mailed.

### 2.2 Alpha testing and revision

An iterative process of reviewing and revising was followed to develop the version of the patient decision aid that is ready for field testing. The first draft was evaluated by a patient communication expert with experience in increasing readability of booklets, leaflets and websites of our institution.

The second draft was sent to a patient expert panel consisting of six *BRCA1/2* mutation carriers who had one or more consultations at our tertiary hospital and had already undergone RRSO. They agreed to get invited for any *BRCA‐*related research projects at the time they participated in a previous study.^8^ Although they do not belong to the ultimate target population, we invited these women to assess the decision aid for two reasons: first, they know the consequences of salpingo‐oophorectomy from their own experience and second, we considered it unethical to offer a preliminary version of the decision aid to women who still had to make a choice for either strategy. They were asked to fill out a questionnaire on structure, content, layout, length, comprehensibility, relevance, credibility and usability of the patient decision aid that was (e)mailed to them. The questionnaire contained 21 questions, of which 13 were open‐ended, and was previously used in the development of another patient decision aid.^25^ Additionally, the decision aid was discussed during an individual audio‐recorded telephone interview to let them tell all their thoughts about content, wording and presentation to the interviewer.

Based on the received feedback, a third draft was developed and alpha‐tested. Forty‐four *BRCA1/2* mutation carriers who had undergone pre‐menopausal RRSO in our hospital between 2010 and 2015 were sent an invitation letter and informed consent form. Names and addresses were retrieved from our institutional operation room registration system. A reminder was sent after 1 month in case of no response. Women who consented to participate were sent the decision aid and a short questionnaire with 12 Likert scale questions, 12 multiple‐choice items and two opportunities for suggestions. Questions were about basic demographics, content, structure, length, balance, comprehensibility, completeness and usefulness. The main difference with the questionnaire in the previous test round was a lower number of open‐ended questions. Again, the questionnaire was based on a questionnaire previously used.^25^ Participants who did not return their questionnaires were reminded three to six times by email and phone. More attempts were found to be undesirable and unethical because it could be too disturbing and might prejudice the voluntary character of study participation. Furthermore, feedback on the decision aid was also requested from patient advocates.

As suggested by Coulter et al,^23^ clinicians were invited to participate in alpha testing as well. A revised fourth draft together with an adjusted questionnaire was emailed to an expert panel of 14 health‐care professionals from ten hospitals throughout the country who counsel participants about ovarian cancer risk‐reducing options in the national preference trial. None of them were involved in the initial development of the decision aid. Although the target audience consists of patients and not clinicians, these professionals were asked to assess the decision aid for accuracy, balance and presentation of information. They were also asked whether they would be willing to hand out this decision aid to their patients. Their suggestions were incorporated in the final version that is ready for field testing.

Based on the study design and confirmed by the Medical Ethical Committee “CMO Regio Arnhem‐Nijmegen,” the Medical Research Involving Human Subjects Act (Dutch: WMO) is not applicable to this study. Therefore, the study was exempted from being appraised by a medical ethical committee.

## 3 RESULTS

### 3.1 Development

#### 3.1.1 Patients’ decisional needs

From our qualitative study, we identified the following factors important in the decision for salpingo‐oophorectomy or salpingectomy with delayed oophorectomy: ovarian and breast cancer risks; onset of premature menopause; level of evidence for the efficacy and safety of either strategy; medical history; family history; number of operations.^8^ The additional literature search yielded two publications on *BRCA1/2* mutation carriers^9^ and professionals^26^ surveyed about salpingectomy with delayed oophorectomy. Factors reported to influence the decision are consequences of premature menopause, HRT, surgical morbidity, potential ovarian damage and lack of data on level of benefit and cancer risks.^9^, ^26^

#### 3.1.2 Content and format

Search results for domains (1) risks and (2) benefits of no surgery, salpingo‐oophorectomy and salpingectomy with delayed oophorectomy are described elsewhere.^24^ Risk estimates based on the literature were incorporated into the decision aid. The literature search for the third domain concerning premature menopause and HRT yielded 79 publications. Two relevant articles outlining all aspects of premature menopause and HRT in *BRCA1/2* mutation carriers were identified and used in the decision aid.^27^, ^28^ Critical appraisal of the level of evidence of selected publications (fourth domain) resulted in how conclusively information was presented. The format of the value clarification worksheet (fifth domain) was based on a systematic review^29^ and the experience within our project group.^25^, ^30^

Analogous to another decision aid,^30^ ours was subdivided into a general introduction, three chapters and a step‐by‐step plan containing a personal value clarification worksheet. Chapter 1 outlines the three options in ovarian cancer risk management with the main risks and benefits. Chapter 2 contains more specific information on (estimated) ovarian and breast cancer risks for the three options. Chapter 3 goes into menopause and HRT. The step‐by‐step plan contained 9 steps to guide the user which information should be read, which individual values matter most and whether one is well‐prepared and confident enough to decide. To elicit one's preferences, eight statements were selected based on patients’ decisional needs and had to be scored for (dis)agreement on a 6‐point Likert scale (3‐point Likert scale in the initial version). Subsequently, the user is asked to rank the three most important statements to clarify which values matter most; however, first drafts contained a rating instead of a ranking exercise. Finally, implications of their stated values for their decision are explicitly shown.

### 3.2 Alpha testing and revision

#### 3.2.1 Alpha testing round 1

The first draft was changed according to the patient communication expert's comments. Wording and sentence structure were adjusted to increase readability. Some detailed medical information was removed because it was thought to be too difficult to understand for the average user. Thereupon, 5 of 6 invited *BRCA1/2* mutation carriers consented to participate in alpha test 1. They filled out a questionnaire and audio‐recorded telephone interviews were subsequently transcribed by one of the authors (M.H.). Their comments led to several major changes. An image of the anatomy of the female internal genitals was added, as was an overview of options with their main (dis)advantages. Participants preferred icon arrays and pie charts for risk communication, so risk tables, which were found confusing, were deleted. Limited alterations were made in wording, text order and colour use which resulted in the third draft.

#### 3.2.2 Alpha testing round 2

Twenty‐five of 44 eligible women (57%) signed and returned the informed consent form for participation in the next round that involved the third draft, two declined and 17 responded neither to our letter nor to a reminder. Nineteen of 25 women (76%) who consented filled out the semiquantitative questionnaire. Four did not return their questionnaires in spite of three to six reminders by phone and email, one withdrew her consent and one was physically not able to complete the questionnaire. Baseline characteristics of the 19 participants are presented in Table 1. Mean age at RRSO was 42.3 years and 14 (73.7%) carried a *BRCA1* mutation. Twenty per cent were breast cancer survivors, and 42% were higher educated.

**Table 1:** Baseline characteristics of *BRCA1/2* mutation carriers who had already undergone risk‐reducing salpingo‐oophorectomy and participated in alpha test round 2

	N = 19
Current age (years, mean ± SD)	45.6 ± 3.878 (Range 37‐51)
Age at RRSO (years, mean ± SD)	42.3 ± 3.603 (Range 34‐48)
Mutation (n)	
* BRCA1*	14 (73.7%)
* BRCA2*	5 (26.3%)
Previous breast cancer (n)	
* *Yes	4 (21.1%)
* *No	15 (78.9%)
Level of education (n)	
* *Primary/pre‐vocational school	2 (10.5%)
* *Vocational education	7 (36.9%)
* *Pre‐college education	2 (10.5%)
* *College/university	8 (42.1%)

RRSO, risk‐reducing salpingo‐oophorectomy.

For chapters 1 and 2 about the three options and (estimated) ovarian and breast cancer risks, the content was judged “good” or “excellent” on a 4‐point Likert scale by at least 95% and presentation of information was judged “good” or “excellent” by at least 89%. Both content and presentation of information in chapter 3 were scored “good” or “excellent” in 84% (menopause part) and 74% (HRT part). Results of other items surveyed are presented in Table 2. Length and the amount of information were assessed “just right**”** by the majority of participants. Including more information about menopause and about risks and prognosis of ovarian cancer was suggested. Furthermore, the information was mainly judged as balanced, realistic and comprehensible, and 79% would find it useful in decision making if they would have to choose between salpingo‐oophorectomy and salpingectomy with delayed oophorectomy. The preference elicitation tool of the personal worksheet was the most criticized: 32% either wanted to add, remove or change some of the statements. No differences were observed between women with and without breast cancer history. In general, patient reviewers were very positive about the clear language and images. The decision aid provided readers with easy‐to‐read information to get an overview of all relevant matters. In contrast, suggestions for improvement were mainly related to information on menopause and HRT, and statements in the preference elicitation tool. A revised version of the decision aid was based on all comments of these 19 patients and four patient advocates. In addition to some textual changes, major changes included revision of chapter 3 (menopause and HRT), adjustment of statement order in the personal worksheet, rephrasing five statements and replacing one statement. Furthermore, the initial rating exercise was replaced by the ranking exercise at this stage. Moreover, we added more detailed information about ovarian cancer prognosis and about the (estimated) risk reduction by the two strategies to chapter 1.

**Table 2:** Alpha testing among *BRCA1/2* mutation carriers and their health‐care providers

	Draft 3*BRCA* mutation carriers (n = 19)	Draft 4Health‐care providers (n = 10)
Length of PtDA, n (%)		
Too long	1 (5%)	7 (70%)
Too short	2 (11%)	0
Just right	16 (84%	2 (20%)
Missing	0	1 (10%)
Amount of information, n (%)		
Too much	1 (5%)	1 (10%)
Too little	6 (32%)	1 (10%)
Just right	12 (63%)	3 (30%)
Missing	0	5 (50%)
Information balanced?, n (%)		
Yes	15 (79%)	8 (80%)
Slanted towards RRSO	3 (16%)	0
Slanted towards RRS/RRO	1 (5%)	2 (20%)
PtDA is comprehensible, n (%)		
In general	17 (89%)	N/A
Risk communication		
Yes	17 (89%)	4 (40%)
No	2 (11%)	4 (40%)
Missing	0	2 (20%)
Consequences of RRSO, n (%)		
Realistic	16 (84%)	9 (90%)
Underestimated	3 (16%)	1 (10%)
Consequences of RRS/RRO, n (%)		
Realistic	13 (68%)	10 (100%)
Underestimated	5 (27%)	0
Overestimated	1 (5%)	0
Useful in decision making, n (%)		
Yes	15 (79%)	8 (80%)
No	3 (16%)	0
Missing	1 (5%)	2 (20%)
Sufficient information to decide, n (%)		
Yes	16 (84%)	8 (80%)
No	3 (16%)	1 (10%)
Missing	0	1 (10%)
Personal worksheet statements, n (%)		
Are well chosen	12 (63%)	6 (60%)
At least one needs to be removed	5 (27%)	1 (10%)
At least one needs to be added	1 (5%)	0
Missing	1 (5%)	3 (30%)
Willing to offer PtDA to patients, n (%)		
Yes	N/A	8 (80%)
No, not this PtDA	N/A	1 (10%)
No, no PtDA at all	N/A	0
Missing	N/A	1 (10%)

PtDA, patient decision aid; RRSO, risk‐reducing salpingo‐oophorectomy; RRS/RRO, risk‐reducing salpingectomy with delayed risk‐reducing oophorectomy; N/A, not applicable.

#### 3.2.3 Alpha testing round 3: professionals

Ten of 14 (71%) professionals from eight hospitals then reviewed the fourth draft of the decision aid and filled out the questionnaire: eight gynaecologic oncologists, one gynaecologist and one medical doctor involved in counselling and supportive care of *BRCA1/2* mutation carriers. For chapters 1 and 2 about the three options and (estimated) cancer risks, both content and presentations were judged “good” or “excellent” on a 4‐point Likert scale by at least 70% of professionals, except for the presentation of HRT that was scored “good” or “excellent” by 60% and “moderate” by the remaining 40%. The majority (70%) found the decision aid too long. Five suggested to develop two separate booklets for *BRCA1* and *BRCA2*. Another concern for half of the responders was the lack of comprehensibility of the presented risks. However, the vast majority classified the information as balanced and realistic, estimated that it would be a useful tool for *BRCA1/2* mutation carriers and confirmed to be willing to offer this decision aid to their patients additionally to in‐person counselling (Table 2). One gynaecologic oncologist was not willing to offer this decision aid to patients because it was too lengthy.

**Figure 2:**
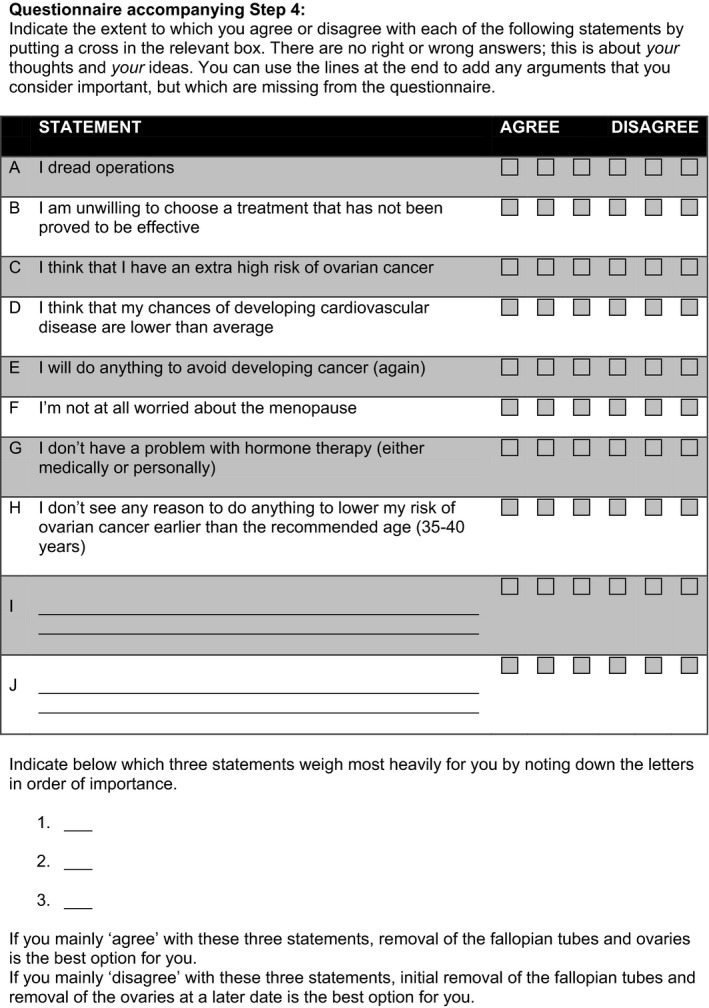
Personal value clarification worksheet of final draft (translated from Dutch into English)

Besides some textual revisions, three significant changes were incorporated into the ultimate version of the decision aid. First, we developed separate decision aids for *BRCA1* and *BRCA2*. Second, examples to estimate ovarian cancer risk were more explicitly described. Third, non‐hormonal options to alleviate effects of premature menopause were added to chapter 3. Figure 2 shows the personal value clarification worksheet (translated from Dutch into English). The final version for *BRCA1* is added as supporting information (S1, in Dutch, could be translated into English upon acceptance).

#### 3.2.4 IPDAS criteria

Quality of the patient decision aid was tested against the 64 IPDAS criteria. As none of the additional criteria were applicable, we checked it against the 50 “regular” items and 37 were satisfied. Among the 23 criteria for “Content,” only criterion 3.9 was not entirely met the following: whether the decision aid allows the user to view probabilities based on their own situation (eg age). The decision aid provides one example applicable to a certain age. We developed tables with risks for various ages^24^ but those were found to be too complicated for this decision aid. However, they are available for health‐care providers and can be discussed during face‐to‐face contact. Fifteen of 20 criteria in the “Development Process” were met. We did not use a readability score to verify readability (criterion 10.4). In addition, we have not field‐tested the decision aid yet (criteria 1.4‐1.5), although we did alpha‐test it. Therefore, criteria regarding the results of field testing were not satisfied the following: whether it is acceptable (criteria 1.6‐1.7), balanced (criterion 9.3) and can be understood by those with limited reading skills (criterion 10.6). Nevertheless, it scored high on the first two criteria during alpha testing. The decision aid was not evaluated by patients with low health literacy, although it was by one dyslectic patient. Lastly, the seven criteria for “Effectiveness” could not be assessed because we have not field‐tested the final version yet.

## 4 DISCUSSION

This paper describes the systematic development process of a paper patient decision aid for ovarian cancer risk management in pre‐menopausal *BRCA1/2* mutation carriers, in close collaboration with *BRCA1/2* mutation carriers and their health‐care professionals. The decision aid elaborates on the risks and benefits of no surgery, RRSO or salpingectomy with delayed oophorectomy. It can be used additionally to face‐to‐face consultation. The last version is found to be clear, complete, balanced and usable by the vast majority of reviewers. It fulfils 37 of 43 quality requirements for content and development process as formulated in the IPDAS checklist. Patient reviewers who underwent risk‐reducing surgery before would have liked to use the decision aid if they would have to decide now.

The systematic development in a multidisciplinary team and the input of patient and professional experts are the main strengths of this study. Furthermore, four patient advocates of a national patients’ association also reviewed the decision aid. The last strength is that we present the user with the implications of their expressed values in the personal worksheet, supposedly leading to better outcomes when using value clarification methods.^29^

The most important limitation in the development of this patient decision aid is the lack of (univocal) evidence for some items. First, salpingectomy with delayed oophorectomy has not been clinically investigated yet, so its effect on ovarian cancer risk is unknown. Second, health consequences of premature menopause in this particular population has not been entirely unravelled and the same applies to the effect of HRT. However, there are no convincing arguments to expect differences between *BRCA1/2* mutation carriers and the general population regarding effects of premature menopause. Furthermore, existing evidence only provides relative risks, which are very hard to be interpreted. For instance, a two‐ to four‐fold increase in the risk of cardiovascular disease is very hard to translate into absolute risks without knowing the background risk, which depends on lifestyle, personal and family medical history etcetera. Furthermore, we did not alpha‐test the decision aid in the actual target population because lack of experience with premature menopause and because of ethical reasons. For the first reason, we neither invited women who underwent only a salpingectomy before, although that could have revealed additional issues to take into account in decision making. Women with low literacy skills were under‐represented in both alpha tests: a common problem in this kind of research that we were not able to resolve.^14^ In addition, we were not able to check our patient decision aid against a standard readability score because no widely used and validated readability score is available for Dutch texts. Readability scores for English do not apply to Dutch.

Two randomized controlled trials evaluating patient decision aids for women at increased risk of ovarian cancer only included RRSO and ovarian screening as options.^15^, ^16^ One also contained information on breast cancer risk management options besides those for ovarian cancer.^15^ All together, they found that women who used the decision aid felt better informed and were more satisfied with the amount and quality of received information compared with women who received usual care. Furthermore, users’ risk estimates for ovarian cancer were more accurate and they chose risk‐reducing surgery more often. However, no statistically significant differences were found for well‐being and decision‐related outcomes, possibly because of lack of power. Tiller et al^16^ developed their decision aid for women with a family history of breast and/or ovarian cancer or Lynch syndrome. Besides ovarian screening and RRSO, information about watchful waiting and chemoprevention was included. Women who used the decision aid reported higher acceptability and the received information as more sufficient and helpful in decision making compared with women receiving a general educational pamphlet. Greater knowledge and lower decisional conflict were found 2 weeks after the intervention but did not last after 6 months. Use of the decision aid did not affect psychological outcomes.^16^ Besides, several decision aids on management options of breast cancer risk in *BRCA1/2* mutation carriers have been developed and tested.^31^, ^32^, ^33^, ^34^ Women who used these decision aids also reported less decisional conflict, reduced uncertainty and cancer‐specific distress, increased knowledge and more satisfaction with their decision.^31^, ^32^, ^33^, ^34^

In conclusion, our systematically developed patient decision aid for pre‐menopausal *BRCA1/2* mutation carriers participating in a Dutch clinical preference trial (NCT02321228) appears to be acceptable and usable according to both patients and professionals. However, whether its use really lowers decisional conflict, increases patient satisfaction and results in informed decisions that are congruent with personal values has to be studied yet. Moreover, the rapidly growing body of evidence for this particular population will require regular updates of the content of the patient decision aid.

## CONFLICT OF INTEREST

All authors declare to have no competing interests.
