# Supplementary material for: A patient decision aid for risk‐reducing surgery in premenopausal BRCA1/2 mutation carriers: Development process and pilot testing
Source: Health Expect. 2017 Dec 27;21(3):659–67. doi: 10.1111/hex.12661 (PMC5980589; doi:10.1111/hex.12661)
Supplement: Supplementary file 1 [file HEX-21-659-s001.pdf]

Decision aid

Preventing ovarian cancer in BRCA1 mutation carriers

Department of Obstetrics and Gynaecology

Radboudumc Nijmegen

September 2017

Introduction

You have a *BRCA1* gene mutation, which means that you have a higher than average risk of developing ovarian cancer. You must decide whether you want to undergo a procedure that will reduce your risk of ovarian cancer. In addition, you must decide *which* procedure to have, and when. These are very difficult decisions; each option has its own advantages and disadvantages. The final decision is up to you, and you will possibly seek help from your next of kin. The doctor treating you can also help. This decision aid provides you with information about the various procedures that are available, and may help you to make up your mind. It explains the main advantages and disadvantages of each procedure, enabling you to weigh them up before deciding which course of action to follow. Although the decision aid may guide you in the direction of a particular procedure, you are obviously free to decide which procedure you think is best for you.

Using the decision aid
The decision aid comprises different sections.

Part 1: Factual information

1) The situation and treatment options
2) The risk of ovarian and breast cancer
3) The menopause

Part 2: A step-by-step plan to help you decide which option suits you best

Section 1. The situation and treatment options
Having a *BRCA1* mutation increases your chances of developing breast cancer and ovarian cancer. Even now, it is impossible to detect ovarian cancer at an early stage, which means that it is usually discovered too late to start adequate treatment. Most patients with advanced ovarian cancer die within 5 years despite undergoing comprehensive treatment. The mortality rate is approximately 60%. If ovarian cancer is caught at an early stage, a maximum of 25% of patients die within 5 years. The only way to lower the risk of developing ovarian cancer is to have surgery to remove your fallopian tubes and ovaries. This is the advice given in the current medical practice guidelines. But we can now offer an alternative to this standard procedure, which includes the possibility of postponing the menopause. Both the current standard procedure and the new alternative procedure result in permanent sterilisation. You must therefore be sure that you do not want any more children, as you will no longer be able to become pregnant. The details of the various methods are given below.

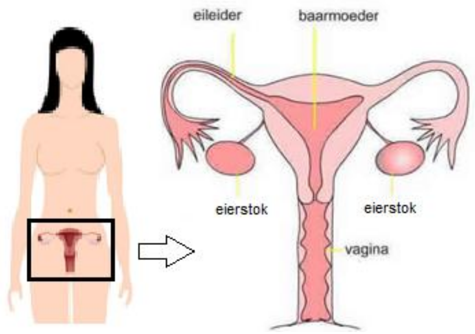

Figure 1. Anatomy of the female internal reproductive organs

What are your choices?
<u>a) You may decline surgery on your fallopian tubes and ovaries to reduce the risk</u>
Advantages:
- No operation(s), no anaesthetic, no risk of post-operative complications.
- You will not have an induced early menopause.

Disadvantages:
- You will still have the higher risk of developing ovarian cancer associated with the *BRCA1* mutation (see page 5).

<u>b) Simultaneous removal of the fallopian tubes and ovaries (current method)</u>
The advice currently given to *BRCA1* mutation carriers in the Netherlands is to have the fallopian tubes and ovaries removed at the age of 35 to 40 years. The operation is usually performed as day surgery. This means that you are admitted to hospital in the morning, undergo surgery and return home the same evening. You will obviously stay in hospital for a night if this is deemed necessary after the procedure. In principle, this is keyhole surgery. The fallopian tubes and ovaries are removed through 3 or 4 small incisions in your abdomen. People with no further health problems can expect a full recovery within 2 weeks. However, if the surgeon is unable to perform the procedure with keyhole surgery, a larger incision is made in the abdomen. Recovery from this type of surgery usually takes longer: approx. 2 to 6 weeks.
Advantages:
- This operation significantly lowers your risk of ovarian cancer (by 80-96%, see section 2).

Disadvantages:
- As with any operation, this procedure involves a risk of specific complications (e.g. haemorrhage, infection and damage to the intestines or urinary tract).
  The risk in this case is relatively small (1 to 3 in every 100 operations). In addition, the effect of the complications is usually temporary and not serious.
- Your menopause will start immediately after the operation (see section 3 for the consequences of an induced early menopause).

c) <u>Removal of the fallopian tubes while postponing removal of the ovaries (alternative method)</u>
As this is a new method, the long-term effects have not been properly researched. Since 2000, there has been strong evidence to suggest that most ovarian cancer starts in the fallopian tubes rather than in the ovaries themselves. For this reason, removing the fallopian tubes at a young age would appear to be a good way of preventing cancer. Women must be sure that they do not want any more children, because once the fallopian tubes are removed, it is impossible to become pregnant naturally. The operation itself is similar to the procedure for removing the ovaries and fallopian tubes simultaneously (see heading b) or possibly less invasive as only the fallopian tubes are removed.
It is still not known whether removing the fallopian tubes really does reduce the risk of ovarian cancer (see section 2), which is why another operation is performed later (at between 40 and 45 years of age) to remove the ovaries.
Advantages:

- The menopause is postponed because the ovaries are left in place for longer.
- You can have an operation before the age of 35, which may lower your risk of ovarian cancer (by an estimated 65%, but this has not yet been proved).

Disadvantages:

- The risk of two operations and the associated complications (e.g. haemorrhage, infection and damage to the intestines or urinary tract).
  The risk of complications from the first operation is small (1 to 3 in every 100 operations). However, scarring in the abdomen from the first operation may make the second operation more difficult. We do not know whether this leads to more complications during the second operation. The effect of the complications is usually temporary and not serious. The doctor treating you may advise against two operations for medical reasons.
- It has not yet been proved that removing the fallopian tubes (without removing the ovaries) reduces the risk of ovarian cancer. Therefore, you might be at higher risk of developing ovarian cancer than if you choose the current standard method, as your ovaries will remain in place for longer. This risk depends on your age. If removal of the fallopian tubes does not reduce the risk, we estimate that of every 100 *BRCA1* mutation carriers who postpone having their ovaries removed from 40 to 45 years of age, approximately an extra 2 will develop ovarian cancer.

A summary of the three options

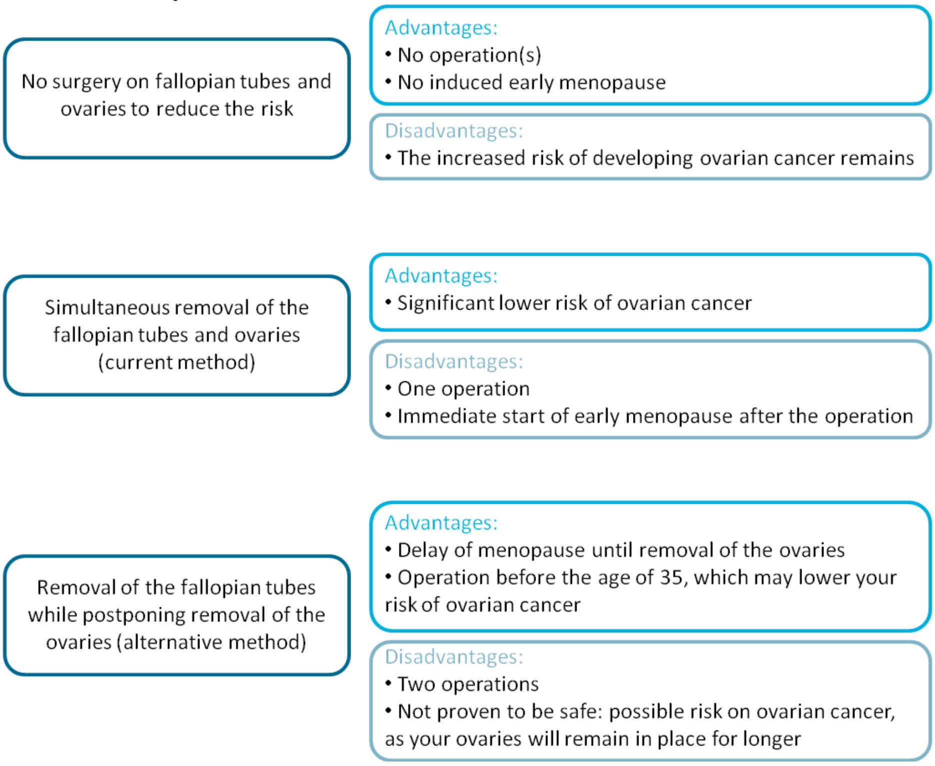

Figure 2. Overview of the three methods and the main advantages and disadvantages

Section 2. The risk of ovarian and breast cancer

2.1. Ovarian cancer

Unfortunately, regular screening of the ovaries using ultrasound and blood counts (CA-125) is not enough to detect ovarian cancer at an early stage and is therefore pointless. The following section explains the effect of various options on your risk of ovarian cancer. Ovarian cancer is also taken to mean cancer of the fallopian tubes and peritoneal cancer.

a) Declining surgery on the fallopian tubes and ovaries to reduce the risk

Ovarian cancer caused by a mutation in *BRCA1* is very rare before the age of 40 (approx. 3 in 100).

***BRCA1* mutation carriers at 40**

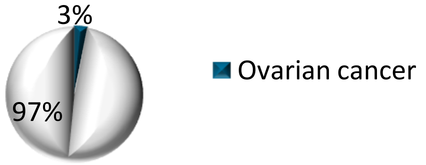

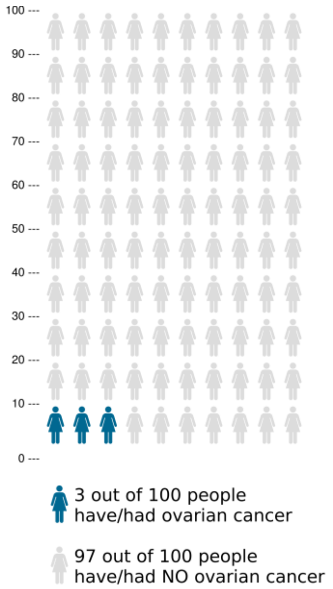

Approximately 39 of every 100 *BRCA1* mutation carriers will develop ovarian cancer before the age of 70.

***BRCA1* mutation carriers at 70**

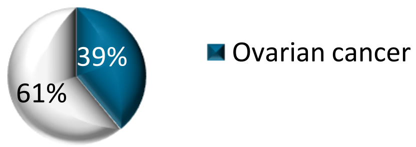

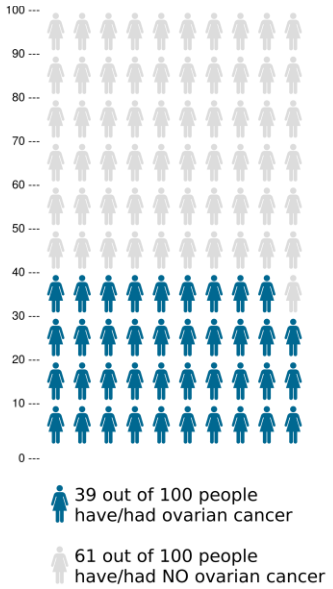

b) Simultaneous removal of the fallopian tubes and ovaries (current method)

Removing the fallopian tubes and ovaries significantly lowers your risk of ovarian cancer. However, ovarian cancer can also occur in the peritoneum. We cannot remove the peritoneum, so there will always be a slight risk of developing peritoneal cancer. The extent of the risk depends on your age. You will find an estimate on page 6.

c) Removal of the fallopian tubes while postponing removal of the ovaries (alternative method)

Nobody knows for sure whether removing the fallopian tubes without removing the ovaries actually lowers the risk of ovarian cancer. There is increasingly more evidence to suggest that a large proportion of ovarian cancers develop in the fallopian tubes. This is particularly true of a certain type, which accounts for approximately 65% of all ovarian cancers developed by *BRCA* mutation carriers. So in 65 of every 100 cases, it may be possible to prevent ovarian cancer by removing the fallopian tubes alone. The other types of ovarian cancer are not as common and less is known about how they develop. It is possible that the fallopian tubes also play a role in the development of other types of ovarian cancer.

We think that the earlier the fallopian tubes are removed, the lower the risk of developing ovarian cancer. As the ovaries remain in place, we cannot be entirely sure that ovarian cancer will not develop in the period between removing the fallopian tubes and removing the ovaries.

Examples

Examples of the two methods aimed at lowering your risk of ovarian cancer are shown below. All examples are based on a *BRCA1* mutation carrier currently at the age of 30. You can read how each method affects the risk of developing ovarian cancer by the age of 70. 'Ovarian cancer' is also taken to mean cancer of the fallopian tubes and peritoneal cancer.

Example 1 shows an estimate of the risks after the simultaneous removal of the fallopian tubes and ovaries, based on previous research. Example 2 shows an estimate of the risks assuming that fallopian tube removal (salpingectomy) reduces the risk of ovarian cancer. As there has not yet been any research into this alternative method, we have estimated the risks on the basis of the data currently available. Example 3 shows an estimate of the risks assuming that fallopian tube removal does *not* reduce the risk of ovarian cancer, and the ovaries remain in place for 5 years longer than in example 1. As you can see, the risk of developing ovarian cancer rises from 3 to 10 in every 100 to 4 to 11 in every 100 if the operations are performed at the age stated in the examples.

Example 1
Current method:

the 35-year-old *BRCA1* mutation carrier has her fallopian tubes and ovaries removed at the age of 38, reducing her risk of ovarian cancer by 80-96%.

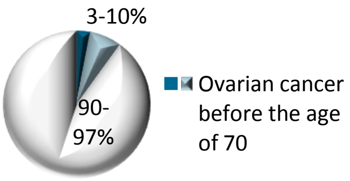

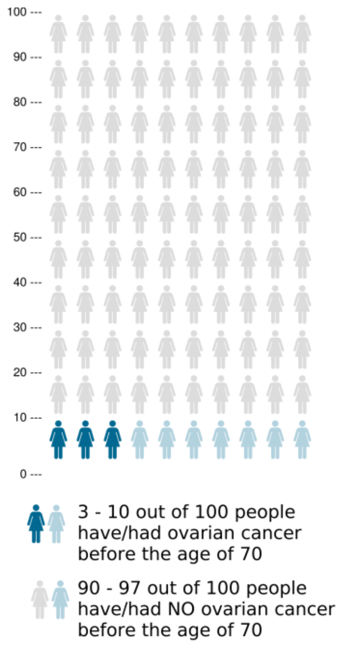

Example 2
Alternative method WITH the effect we would expect:

the 35-year-old *BRCA1* mutation carrier has her fallopian tubes removed at the age of 38, reducing her risk of ovarian cancer by 65%. She then has her ovaries removed at 43, reducing the risk even further.

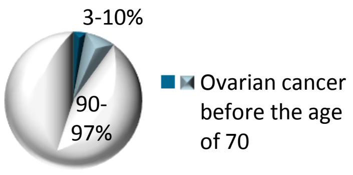

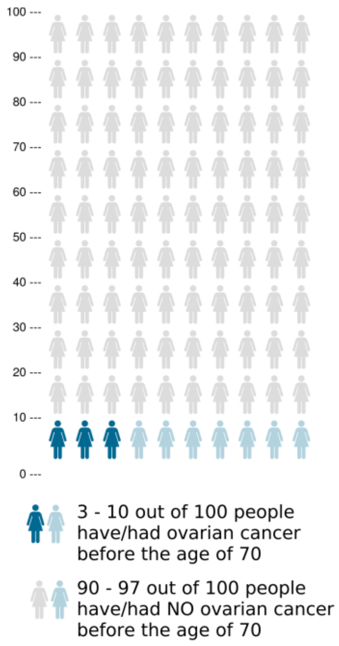

Example 3
Alternative method WITHOUT the effect from salpingectomy:

the 35-year-old *BRCA1* mutation carrier has her fallopian tubes removed at the age of 38, with no effect on her risk of ovarian cancer. She then has her ovaries removed at 43, which does reduce the risk. This risk corresponds with the risk she would have had if her fallopian tubes and ovaries were removed simultaneously at the age of 43.

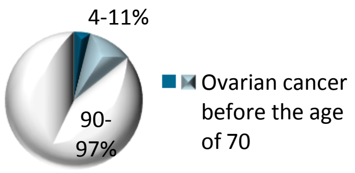

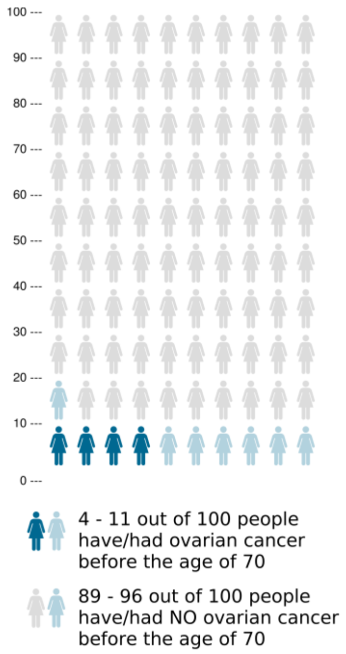

__2.2. Breast cancer__

The following section is about the risk of breast cancer and how the various treatment options affect this risk.

Women who have undergone a double mastectomy (the surgical removal of both breasts) to prevent breast cancer have no risk of developing breast cancer.

a) Declining surgery on fallopian tubes and ovaries (and no mastectomy)

Approximately 12 in every 100 *BRCA1* mutation carriers develop breast cancer by the age of 40.

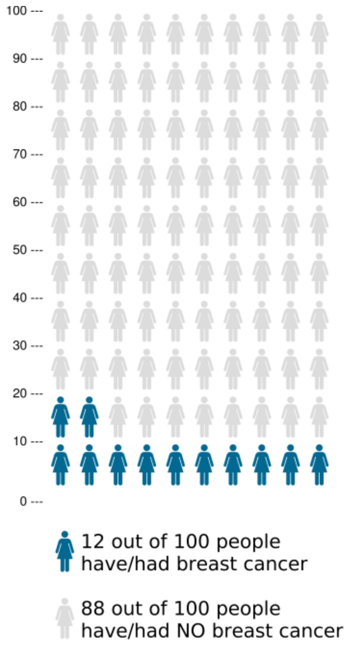

Approximately 60 in every 100 *BRCA1* mutation carriers develop breast cancer by the age of 70.

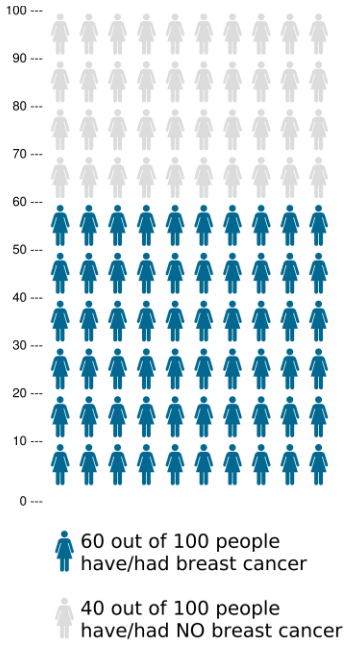

b) Simultaneous removal of the fallopian tubes and ovaries (current method)

Removing the fallopian tubes and ovaries probably does not affect the risk of breast cancer. However, until 2015, it was thought that the risk of developing breast cancer would be halved if the fallopian tubes and ovaries were removed before the natural menopause. This does not seem to be the case. So you do not need to consider the risk of developing breast cancer when deciding whether to have your fallopian tubes and ovaries removed to reduce the risk of ovarian cancer.

c) Removal of the fallopian tubes while postponing removal of the ovaries (alternative method)

Removing the fallopian tubes (the first operation) does not reduce the risk of breast cancer. Regarding the second operation, the same applies as in b): it is highly improbable that removal of the ovaries at a later date will reduce the risk of breast cancer.

Section 3. Menopause

a) Declining surgery on fallopian tubes and ovaries

The average age at which Dutch women start the menopause is 50-51 years. This is the stage in life when hormone production in the ovaries drops and eventually all but stops. As a result, women stop menstruating and develop symptoms of the menopause. The menopause is usually a gradual process. Some women hardly notice the changes, while others have symptoms that have a negative impact on their quality of life.

Typical symptoms of the menopause: Hot flushes are the most common complaint, but women also experience fatigue, mood swings, joint pain, skin changes and altered body perception. It is difficult to predict how the menopause will affect individual women as it varies greatly from person to person.

Sexuality: The low levels of female hormones often mean that women are less interested in sex (medical term: loss of libido). In addition, the mucous membranes inside the vagina can become drier, which can lead to pain during intercourse.

Osteoporosis: The lack of hormones after the menopause means that less bone is made, causing a drop in bone mass. This high risk of osteoporosis increases the risk of bone fractures.

Cardiovascular disease: The lack of female hormones after the menopause increases the risk of cardiovascular disease, and ultimately death.

b) Simultaneous removal of the fallopian tubes and ovaries (current method)

If both ovaries are removed, almost all female hormones disappear from the body. The woman goes through an induced menopause 'overnight', 10 to 15 years earlier than she would have under normal circumstances (assuming the recommended age for the removal of the fallopian tubes and ovaries). The symptoms and effect of the menopause mentioned above occur immediately after the operation, therefore earlier than normal. At 50, women who have gone through the menopause 10 years earlier are two to four times more likely to develop cardiovascular disease. The extent of the risk depends on several other factors, which is why we cannot be more precise. Finally, there would appear to be link between early-onset menopause and dementia. Hormone therapy can help to counter the adverse effects.

Hormone therapy:

In principle, the advice is to use hormone therapy until around the age of 50. Hormones can be administered as tablets or plasters in combination with an IUD containing hormones. However, not all women want (or are able) to take hormones. It is not recommended for women who have had breast cancer, for example. The usual procedure is to weigh up your options in consultation with a doctor. It should also be said that the effect of hormone therapy varies. It can take some time before a woman finds the medication that suits her best. In addition, the effect of hormone therapy has not been proved for all the consequences of the menopause. Typical symptoms and problems of a sexual nature often improve, but are never completely resolved. Hormone therapy probably prevents osteoporosis to a large extent, and it possibly has a beneficial effect on cardiovascular disease and dementia, but this still needs to be clarified. The link between hormone therapy and Parkinson's disease is still uncertain.

Non-hormonal therapy:

If you do not want hormone therapy or if you are advised against it, there are other non-hormonal options that will reduce your symptoms and the long-term effects. They include life-style advice (diet, exercise, not smoking), as well as non-hormonal medicines or medicines to help prevent osteoporosis (e.g. vitamin D).

c) Removal of the fallopian tubes while postponing removal of the ovaries (alternative method)

Removing just the ovarian tubes has little or no effect on the production of hormones. In principle, removing the fallopian tubes without removing the ovaries does not induce the menopause. You will continue to menstruate. You will, however, go through the menopause when your ovaries are removed later (possibly still earlier than in normal circumstances). You may still be eligible for hormone therapy as described in b), depending on your age.

STEP-BY-STEP PLAN

The following step-by-step plan is designed to help you make the right choice for *you*.

Step 1: Take all the time you need to digest the information and form an opinion. It is important not to rush yourself.

Step 2: Here is a summary of the various treatment options:

- You can decline surgery aimed at lowering your risk of ovarian cancer (this option has now been discounted in this step-by-step plan).
- Simultaneous removal of the fallopian tubes and ovaries at the recommended age. This is between 35 and 40 years old.
- Removal of the fallopian tubes, once your family is complete. Removal of the ovaries is postponed until you are between 40 and 45 years old.

Step 3: Read the information in this decision aid carefully and let it sink in. This also applies to any other information you have received from your doctor. Ask your doctor any questions that occur to you.

Step 4: Summarise the arguments you consider important. The questionnaire on the next page will help you with this.

Step 5: Discuss the information and your thoughts with the people close to you (e.g. partner, relatives, friends).

Step 6: Decide whether you have enough information to make your decision by completing the following questionnaire.

	Yes	No
**1. Do you fully understand the options that are open to you?**<br>**If you answer 'No': Read the information again and/or contact your doctor*	☐	☐*
**2. Are you clear about which advantages and disadvantages of each option are most important to you?**<br>** If you answer 'No': Discuss your thoughts again with your next of kin and/or doctor*	☐	☐*
**3. Have you had enough support and advice to enable you to make a decision?**<br>** If you answer 'No': Discuss your thoughts again with your next of kin and/or doctor*	☐	☐*

Step 7: Which option are you inclined to choose at the moment?

☐ Simultaneous removal of the fallopian tubes and ovaries at the recommended age, i.e. between 35 and 40 years old.

☐ Removal of fallopian tubes, once your family is complete, postponing the removal of the ovaries until you are between 40 and 45 years old.

☐ Do nothing: no surgery on fallopian tubes or ovaries.

Step 8: How certain about your decision are you at the moment?

**Not at all certain**	**0**	**1**	**2**	**3**	**4**	**5**	**6**	**7**	**8**	**9**	**10**	**Convinced**
	☐	☐	☐	☐	☐	☐	☐	☐	☐	☐	☐	

Step 9: What else do you need to help you make your decision?

☐ I'm ready to come to a decision

☐ I still want to discuss my options with others

☐ I'd like more information about the options

Questionnaire accompanying Step 4:
Indicate the extent to which you agree or disagree with each of the following statements by putting a cross in the relevant box. There are no right or wrong answers; this is about *your* thoughts and *your* ideas. You can use the lines at the end to add any arguments that you consider important, but which are missing from the questionnaire.

	STATEMENT	AGREE					DISAGREE
A	I dread operations	☐	☐	☐	☐	☐	☐
B	I am unwilling to choose a treatment that has not been proved to be effective	☐	☐	☐	☐	☐	☐
C	I think that I have an extra high risk of ovarian cancer	☐	☐	☐	☐	☐	☐
D	I think that my chances of developing cardiovascular disease are lower than average	☐	☐	☐	☐	☐	☐
E	I will do anything to avoid developing cancer (again)	☐	☐	☐	☐	☐	☐
F	I'm not at all worried about the menopause	☐	☐	☐	☐	☐	☐
G	I don't have a problem with hormone therapy (either medically or personally)	☐	☐	☐	☐	☐	☐
H	I don't see any reason to do anything to lower my risk of ovarian cancer earlier than the recommended age (35-40 years)	☐	☐	☐	☐	☐	☐
I	______________________________ ______________________________	☐	☐	☐	☐	☐	☐
J	______________________________ ______________________________	☐	☐	☐	☐	☐	☐

Indicate below which three statements weigh most heavily for you by noting down the letters in order of importance.

1. ___
2. ___
3. ___

If you mainly 'agree' with these three statements, removal of the fallopian tubes and ovaries is the best option for you.
If you mainly 'disagree' with these three statements, initial removal of the fallopian tubes and removal of the ovaries at a later date is the best option for you.

This decision aid was developed by the Department of Obstetrics and Gynaecology in the Radboudumc, Nijmegen, The Netherlands. It was developed as part of the TUBA study, funded by the Dutch Cancer Society, project number KUN 2014-7187. The developers, members of the TUBA study project group, have no interest in influencing users to choose either the current or the alternative method. The TUBA study project group would like to emphasise that the decision aid was devised for use in a scientific project, supplementary to a consultation with a gynaecologist. In other words, the decision aid should not be seen as a stand-alone product. A list of the references used to compile the decision aid can be provided on request.

Version 5.0, September 2017

Last update: September 2017

The TUBA study project group consists of:
Project leaders:
Dr J.A. de Hullu, gynaecological oncologist, Radboudumc Nijmegen
Dr R.P.M.G. Hermens, senior researcher/epidemiologist, IQ Healthcare, Radboudumc Nijmegen
Prof. N. Hoogerbrugge, oncogeneticist, Radboudumc Nijmegen

Research physicians:
M.G. Harmsen, Radboudumc Nijmegen
M.P. Steenbeek, Radboudumc Nijmegen

Project group members:
J. in ’t Hout, biostatistician, Radboudumc Nijmegen
Prof. L.F.A.G. Massuger, gynaecological oncologist, Radboudumc Nijmegen

Expert panel:
Eight gynaecological oncologists, a gynaecologist and a specialist in family-related gynaecology, from eight Dutch hospitals.

Reactions to the decision aid ‘Preventing ovarian cancer in *BRCA1* mutation carriers’
The TUBA project group welcomes any comments concerning mistakes in the lay-out or content of this decision aid. Please use the general e-mail address: onderzoektuba.verlgyn@radboudumc.nl
